# Cysteine proteases in protozoan parasites

**DOI:** 10.1371/journal.pntd.0006512

**Published:** 2018-08-23

**Authors:** Jair L. Siqueira-Neto, Anjan Debnath, Laura-Isobel McCall, Jean A. Bernatchez, Momar Ndao, Sharon L. Reed, Philip J. Rosenthal

**Affiliations:** 1 Center for Discovery and Innovation in Parasitic Diseases, Skaggs School of Pharmacy and Pharmaceutical Sciences, University of California San Diego, La Jolla, California, United States of America; 2 National Reference Centre for Parasitology, The Research Institute of the McGill University Health Center, Montreal, Canada; 3 Program in Infectious Diseases and Immunity in Global Health, The Research Institute of the McGill University Health Centre, Montreal, Quebec, Canada; 4 Departments of Pathology and Medicine, University of California San Diego School of Medicine, La Jolla, California, United States of America; 5 Department of Medicine, University of California, San Francisco, San Francisco, California, United States of America; Johns Hopkins Bloomberg School of Public Health, UNITED STATES

## Abstract

Cysteine proteases (CPs) play key roles in the pathogenesis of protozoan parasites, including cell/tissue penetration, hydrolysis of host or parasite proteins, autophagy, and evasion or modulation of the host immune response, making them attractive chemotherapeutic and vaccine targets. This review highlights current knowledge on clan CA cysteine proteases, the best-characterized group of cysteine proteases, from 7 protozoan organisms causing human diseases with significant impact: *Entamoeba histolytica*, *Leishmania* species (sp.), *Trypanosoma brucei*, *T*. *cruzi*, *Cryptosporidium* sp., *Plasmodium* sp., and *Toxoplasma gondii*. Clan CA proteases from three organisms (*T*. *brucei*, *T*. *cruzi*, and *Plasmodium* sp.) are well characterized as druggable targets based on in vitro and in vivo models. A number of candidate inhibitors are under development. CPs from these organisms and from other protozoan parasites should be further characterized to improve our understanding of their biological functions and identify novel targets for chemotherapy.

## Introduction

Proteases are enzymes that catalyze the hydrolysis of peptide bonds and are important in a number of biological activities, including digestion of peptides, activation of other enzymes, modulation of the immune system, participation in the cell cycle, and differentiation and autophagy. There are at least 6 classes of proteases classified according to the nucleophilic group responsible for the first step in the proteolysis: serine, cysteine, metallo, aspartate, glutamate, and threonine proteases. Cysteine proteases (CPs) are categorized into 72 families, but not all are represented in protozoan parasites [1]. The most abundant and well characterized CPs in these organisms are the clan CA papain-family enzymes, named after an abundant protease present in papaya fruit. We selected 7 protozoan organisms with medical relevance for review of the current knowledge regarding their clan CA CPs. Table 1 summarizes well-characterized clan CA CPs from these organisms, and Table 2 lists some well-studied inhibitors.

**Table 1:** CPs of protozoan parasites.

Protozoan parasite	Enzymes	EuPathDB entry	Proposed function	Reference
*Entamoeba histolytica*	EhCP1	EHI_074180	Invasion, virulence factors, induces macrophage proinflammatory response, tight junction proteolysis, degradation of ECM (fibronectin, laminin, collagen)	[8, 10, 16, 18]
	EhCP2	EHI_033710	Invasion, virulence factors, induces macrophage proinflammatory response, tight junction proteolysis, degradation of ECM (fibronectin, laminin, collagen)	[6–8, 10, 16, 18]
	EhCP5	EHI_168240	Invasion, virulence factors, induces macrophage proinflammatory response, degradation of ECM (fibronectin, laminin, collagen), degradation of IgA, IgG, pro–IL-1β, pro–and mature IL-18 (immunoevasion), inactivation of anaphylatoxins	[10–15]
	EhCP7	EHI_039610	Invasion, virulence factors, induces macrophage proinflammatory response	[16, 18]
	EhCP (unspecified)	unspecified	Virulence factor, host defense evasion	[6–8]
	EhCP (unspecified)	unspecified	Parasite encystation–excystation	[17]
*Leishmania* sp.	CPA, CPB1, CPB2	LmxM.19.1420, LmjF.19.1420, LinJ.19.1460; LmxM.07.0550, LmjF.08.1010;	Virulence factor; immunomodulators; parasite metabolism modulator	[26, 33, 35, 37, 149]
	CPC	LmxM.08_29.0820, LmjF.29.0820, LinJ.29.0860	In vitro virulence factor; role in parasite cell death	[42]
	Atg4	LmjF.30.0270, LinJ.30.0270	Autophagy (differentiation and virulence)	[41, 150]
	otubain	LmxM.17.1400, LmjF.17.1400, LinJ.17.1520	Deubiquitination, proinflammatory activity	[42]
*Trypanosoma brucei*	TbCatL (brucipain, rhodesain)	Tb927.6.960-Tb927.6.1060	CatL-like activity, virulence factor, promotes crossing of the BBB, host immune evasion	[51, 54, 151]
	TbCatB	Tb427.06.560	Virulence factor, iron acquisition	[59–61, 152]
*T*. *cruzi*	Cruzain (GP57/51, TcCatL)	TCDM_05847-t26_1	Virulence factor, evasion of immune system, invasion of host cells, parasite differentiation	[2, 3, 73]
	Calpain-like (TcCatB)	TcCLB.506227.130, TCDM_01392	Signaling transduction, metacyclogenesis	[75]
	Atg4	TCDM_05817-t26_1, TCDM_06287-t26_1	Parasitic autophagy important in differentiation and virulence	[76, 153]
*Cryptosporidium* sp.	Cryptopain (CatL-like, CpaCATL-1)	cubi_02618-t31_1, cgd3_680, cgd4_2110, cgd6_4880, cgd7_2850	Host cell invasion	[89, 90]
	CpOTU (otubain)	cgd6_2750	Ubiquitin pathway; peak in the oocyst stage	[93]
*Plasmodium* sp.	Falcipain-1	PF3D7_1458000.1	Oocyst production	[110]
	SERA family. Atypical CP and dipeptidyl aminopeptidase 3.	PF3D7_0207500	Invasion of hepatocytes by sporozoites	[103, 106]
	Falcipain-2, falcipain-3, dipeptidyl aminopeptidase 1	PF3D7_1115700.1, PF3D7_1115400.1	Hemoglobin hydrolysis	[101, 122]
	PfOTU	PF3D7_1031400	Apicoplast development and homeostasis	[115]
*Toxoplasma* sp.	Single copy gene CatB (TgCPB)	TGME49_249670	Cell invasion and replication	[140, 142]
	Single copy gene CatL (TgCPL)	TGME49_321530	Host cell invasion, digestion of cytosolic proteins	[134, 141]
	TgCP1 and TgCP2 (CatC-like), exopeptidases	TGME49_289620, TGME49_276130	Expressed in tachyzoites, degrade peptides	[137]

**Abbreviations:** BBB, blood–brain barrier; CP, cysteine proteases; ECM, extracellular matrix; EuPathDB, Eukaryotic Pathogen Database; sp., species.

**Table 2:** CP inhibitors.

Inhibitor	Targeted enzyme	Organisms/disease	References
K11777	Cruzain, TcCatL, cryptopains, TgCPL, TgCPB, EhCP1, LtCP	*Trypanosoma cruzi*, *T*. *brucei*, *Cryptosporidium parvum*, *Toxoplasma gondii*, *Entamoeba histolytica*, *Leishmania tropica*	[2, 19, 60, 91, 149]
WRR-483	Cruzain, EhCP1	*T*. *cruzi*, *E*. *histolytica*	[19, 82, 154–156]
Cz007, Cz008	Cruzain	*T*. *cruzi*	[81]
Gallinamide A	Falcipain-2, falcipain-3	*Plasmodium falciparum*	[128]
Symplostatin 4	Falcipain	*P*. *falciparum*	[157]
Tetrafluorophenoxymethyl ketone	Cruzain	*T*. *cruzi*	[158]
Odanacatib	Human cathepsin K	osteoporosis	[159]
Balicatib	Human cathepsin K	osteoporosis	[160]
Emricasan	Human caspase-8	Liver protection, leukemia	[161]
SAR114137	Human cathepsin S	Chronic pain	https://ncats.nih.gov/files/SAR114137.pdf
RWJ-445380	Human cathepsin S	Rheumatoid arthritis	https://ncats.nih.gov/files/RWJ-445380.pdf

**Abbreviation**: CP, cysteine protease.

Despite the essentiality of CPs for parasite survival, there are, as yet, no approved drugs targeting this group of enzymes. The most advanced drug in development against a parasite CP is the vinyl sulfone inhibitor K11777, with in vivo efficacy in an animal model of Chagas disease [2, 3]; K11777 is currently moving forward to clinical trials.

### Entamoeba histolytica

*E*. *histolytica* causes amebiasis and is responsible for about 70,000 deaths per year [4, 5]. The parasite infects the intestinal tract and can cause diarrhea, colitis, and peritonitis. Extraintestinal amebiasis can also occur, commonly leading to amoebic liver abscesses. *E*. *histolytica* CPs are encoded by approximately 50 genes and are responsible for epithelial barrier penetration and degradation of host extracellular matrix components. Only four proteases, EhCP1, EhCP2, EhCP5, and EhCP7, are highly expressed by *E*. *histolytica* in culture and have identified cellular localizations. EhCP1 is present in intracellular vesicles, EhCP2 localizes to the internal and external cell membrane, and EhCP5 is on the cell surface. Invasive *E*. *histolytica* clinical isolates have 10×–1,000× more CP activity in culture than noninvasive *E*. *dispar* isolates, suggesting a role for CPs in parasite virulence and invasion [6].

Secreted *E*. *histolytica* CPs cleave the colonic mucus layer and overcome mucosal host defenses in the gut [7]. Villin and tight junction proteolysis are also mediated by these CPs [8]. EhCP1, EhCP2, and EhCP5 have been shown to degrade extracellular matrix components, such as fibronectin, laminin, and collagen [9]. Transcriptional studies identified increased expression of EhCP1 and EhCP2 when *E*. *histolytica* interacted with human collagen and mucin [10]. Those results corroborate the key role of these CPs in parasite invasion of host tissues.

*E*. *histolytica* CPs are also hypothesized to interfere with both innate and adaptive host immune responses. They degrade human IgA, the most abundant immunoglobulin defense at the mucosal surface [11]. Extracellular CPs disrupt the heavy chain of IgG, and this is considered to be a key strategy for *E*. *histolytica* survival during tissue invasion [12]. *E*. *histolytica* CPs activate the alternative pathway of the host complement system and generate hemolytically active C3b, but extracellular CPs circumvent host immunity by degrading and inactivating anaphylatoxins C3a and C5a [13]. *E*. *histolytica* CPs modulate cell-mediated immunity as well. Extracellular CPs degrade pro–IL-1β that is released by damaged intestinal epithelial cells to form the active mature proinflammatory cytokine IL-1β [14]. On the other hand, *E*. *histolytica* CPs cleave both pro–and mature IL-18 [15], and the degradation of IL-18 may contribute to either parasite or host survival. A recent study demonstrated the function of EhCP1, EhCP4, and EhCP5 in the intercellular junction of *E*. *histolytica* and macrophages to modulate the host cell cytoskeleton and trigger subsequent IL-1β secretion, detailing the cascade of molecular events triggered by the initial interaction of *E*. *histolytica* with macrophages [16]. That study suggests new roles for EhCP1 and EhCP4 in inducing a proinflammatory response by host macrophages upon cellular contact. EhCPs may also contribute to parasite encystation–excystation [17] and in nutrient acquisition through erythrophagocystosis [18], both essential to parasite infectivity and survival.

The vinyl sulfone inhibitors K11777 and WRR483 were tested against recombinant EhCP1, and both demonstrated potent inhibitory activity, significantly reducing parasite invasion of human colonic xenografts [19]. Further studies should be carried out to develop an antiamebiasis drug targeting CPs.

### *Leishmania* sp.

Leishmaniasis is a spectrum of diseases, including skin ulcers; degradation of nose and palate mucosal tissue; and liver, spleen, and bone marrow infiltration. This latter visceral form of the disease is the most lethal, killing over 60,000 people per year [20]. The factors determining clinical manifestations are not fully understood; these are strongly influenced by parasite strain or species and by immunological factors [21]. *Leishmania* express a broad range of CPs, the best characterized of which are CPA, CPB, and CPC (CPs A, B, and C), all part of clan CA, family C1.

CPA and CPB are cathepsin L-like and likely show some functional redundancy [22], while CPC is cathepsin B-like. Cathepsin B-like and cathepsin L-like enzymes have key structural differences associated with divergent substrate binding and proteolytic mechanisms. In cathepsin L, binding of the peptide substrates spans the entire channel between the two domains of the protein, leading to endopeptidolytic activity. In contrast, cathepsin B has an additional occlusion loop that restricts substrate binding under low pH, leading to carboxypeptidase activity; this loop is displaced at high pH, at which point cathepsin B displays endopeptidase activity [23, 24].

CPB genes are arranged in tandem arrays [25], with 19 copies in *L*. *mexicana*, 8 in *L*. *major* [26], and 5 in *L*. *chagasi* [27], while CPA and CPC are single copy [28, 29]. CP gene expression is stage regulated; most are expressed at higher levels in the mammalian amastigote stage than in the insect promastigote stage [25, 29]. However, CPB1 and CPB2 are expressed at higher levels in metacyclic promastigotes [30] and CPC in procyclic promastigotes [28]. Consistent with elevated CP expression in the amastigote stage, *Leishmania* CPs play key roles in the interactions between *Leishmania* and its mammalian host. CPA-knockdown or knockout *L*. *chagasi* and *L*. *infantum* show decreased in vitro [31] and in vivo infectivity [32]. Likewise, CPB-knockout *L*. *mexicana* displayed impaired macrophage infectivity and delayed lesion progression [33]. Multiple CPB copies are required for significant restoration of virulence [34]. CPB modulates host responses to *L*. *mexicana* by down-regulating protective Th1 immune responses, and in particular IFN-γ production, via degradation of the transcription factor NF-κB and subsequent inhibition of IL-12 production by infected macrophages [35]. *L*. *mexicana* CPs also cleave JNK and ERK MAP kinases. Both kinases negatively regulate IL-12 production, and therefore the protozoan cysteine protease CPB can alter host macrophage signaling by increasing IL-12 transcription [35]. *L*. *amazonensis* likewise inhibits antigen presentation via CP-mediated degradation of MHC class II molecules [36]. CPB also modulates levels of parasite proteins, including gp63 [37], a major virulence factor in *Leishmania*. Episomal expression of gp63 in CPB-deficient parasites was sufficient to restore virulence to wild-type levels [37]. Several of the effects attributed to CPB via knockout studies may therefore occur via modulation of other parasite proteins rather than through direct cleavage by CPB. CPC, in contrast, is involved in *Leishmania* cell death [38] and secretome remodeling [39]; knockouts were attenuated in macrophage infection in vitro but were nevertheless able to cause lesions in vivo, suggesting compensatory mechanisms [40].

Other less-studied clan CA members in *Leishmania* include Atg4.1 and Atg4.2 (clan CA, family C54) and otubain-like enzymes (clan CA, family C65). Atg4.2 knockout was associated with impaired metacyclogenesis, altered autophagosome accumulation (especially under nutritional stress), increased amastigote length, and impaired amastigote proliferation; in contrast, Atg4.1 knockout only showed minor effects on promastigote infectivity [41]. A novel otubain-like CP is a deubiquitinating enzyme recently characterized from *L*. *infantum* that may also have proinflammatory activity by stimulating lipid droplet biogenesis and inducing IL-6 and TNF-α secretion [42].

Early work with vinyl sulfone and dihydrazide compounds showed successful in vivo treatment of cutaneous leishmaniasis by CPC inhibition [43]. Since then, several other CPC inhibitors have shown in vivo efficacy to treat cutaneous (palladacycle DPPE 1.2 [44]; organotellurane RT-01 [45]) and visceral (organotellurane RF07 [46]) leishmaniasis. In vitro assays identified multiple compound classes active against CPB (e.g., semicarbazones, thiosemicarbazones, triazine nitriles [47], and benzophenones [48]), but these have yet to be tested in vivo.

### Trypanosoma brucei

Human African trypanosomiasis (sleeping sickness), caused by various subspecies of *T*. *brucei*, is found in sub-Saharan Africa. The flagellated parasite invades the blood–brain barrier, causing fatal damage in the central nervous system, with approximately 7,000 deaths per year [20]. Similar to *Leishmania* CPs, the best characterized *T*. *brucei* CPs are cathepsin-like enzymes, cathepsin L (TbCatL, also known as brucipain and rhodesain in different *T*. *brucei* subspecies) and cathepsin B (TbCatB) [49].

TbCatL and TbCatB are both involved in virulence. TbCatL promotes parasite crossing of the blood–brain barrier via activation of host G-protein–coupled receptors such as PAR2 (protease-activated receptor 2) and subsequent induction of host calcium-signaling pathways [50, 51]. TbCatL also protects *T*. *brucei* from lysis by host serum [52, 53] and from opsonization via degradation of internalized variant surface-glycoprotein–bound antibodies [54]. As for TbCatL [55], TbCatB expression is greatest in the bloodstream stage [56]; it mediates the degradation of endocytosed host transferrin in the parasite endosomal/lysosomal compartment as part of *T*. *brucei* iron acquisition pathways [57, 58]. TbCatB may also be involved in cytokinesis [56]. A number of studies using RNA interference (RNAi) [58] or small-molecule inhibitors in vitro [59] and in animal infection models [60, 61] have validated these proteases as drug targets. K11777 in particular targets TbCatL: it showed synergy when combined with eflornithine and might be considered for development as a new therapy for African trypanosomiasis [62]. Other small molecules have been successfully tested against TbCatL, including dipeptide nitriles [63] and triazine nitriles [64], but their efficacy in vivo is yet to be determined. A recent review highlights a thorough list of candidate *T*. *brucei* and *T*. *cruzi* CP inhibitors [65].

### Trypanosoma cruzi

Chagas disease is endemic in the Americas and is the main cause of heart failure in Latin America, leading to more than 12,000 deaths every year [66]. It is caused by the parasite *T*. *cruzi*, which encodes genes from four clans of CPs: CA, CD, CE, and CF. Clan CA includes the most abundant protease of this parasite: cruzain (cruzipain), a papain-like cathepsin L-like member of family C1.

Cruzain is present in epimastigotes and bloodstream trypomastigotes and is identified as a major antigen in infected humans [67], and for that reason, it has been considered as a vaccine candidate [68]. It plays important roles in differentiation [69], metabolism [70], evasion of the immune response, and invasion of host cells [71]. In trypomastigotes (infective stage), cruzain is localized in the flagellar pocket, and in intracellular amastigotes, the enzyme is on the cell surface. Recombinant cruzain was expressed in bacteria and demonstrated protease activity after autocatalytic activation [72]. The crystal structure of cruzain complexed with the inhibitor Z-Phe-Ala-fluoromethyl ketone was resolved, confirming structural similarities with papain.

Genes coding for a 30-kDa cathepsin B-like CP, two other cathepsins, and other homologues of calpains (family C2) are also present [74]. Other members of clan CA include autophagin-like Atg4 protease (family of C54), responsible for processing Atg8 for the formation of the autophagosomes involved in the parasitic autophagy process essential for metacyclogenesis and virulence [75, 76]. Atg4 has recently been proposed as a potential new target for chemotherapy [76].

The idea of developing CP inhibitors as Chagas disease chemotherapy originated in observations of in vitro antiparasitic effects associated with cruzain inhibition. Because of multiple copies of the cruzain gene, gene knockout could not be achieved to confirm the essentiality of the enzyme, even though “chemical validation” with protease inhibitors suggested that it is essential. The first proof of concept in an animal model showed that mice could be rescued from a lethal *T*. *cruzi* infection with the vinyl sulfone inhibitor K11777 dosed for 20 days [3]. Surviving mice had negative hemoculture, indicating parasitological cure. The same inhibitor was later tested in a dog model of Chagas disease, and it prevented cardiomyopathy after 7 days of treatment [2].

A number of other cruzain inhibitors are under study. Computational screening of the ZINC database identified inhibitors with nanomolar potency against cruzain [77]. Some isatins also have the thiosemicarbazone functionality, a well-known cruzain inhibitor group [78]. However, isatin compounds without a thiosemicarbazone also demonstrated inhibitory activity against cruzain [79]. These compounds have a peptide sequence recognized by the catalytic site of cruzain as well as the epoxy electrophile reminiscent of the E-64 inhibitor. Odanacatib, a reversible inhibitor of human cathepsin K with an amino nitrile warhead, was in Phase III trials to treat osteoporosis, but its development was discontinued due to risk of stroke (see the associated chapter, “Cysteine proteases as digestive enzymes in parasitic helminths,” for information on odanacatib efficacy against hookworm infection). The nitrile warhead forms a reversible (albeit with a slow off rate) thioimidate with the active site of human cathepsin K [80]. Inspired by this approach, reversible inhibitors have been developed against cruzain with 100× selectivity compared to human cathepsins L, B, S, and F. Further optimization of these compounds led to the discovery of Cz007 and Cz008, with IC_50_s against cultured parasites in the nanomolar range and antiparasitic efficacy in a mouse model of Chagas disease [81]. More recently, the vinyl sulfone WRR-669 was demonstrated to be a noncovalent inhibitor of cruzain. These results, showing both in vitro and in vivo efficacy against *T*. *cruzi*, support cruzain as a valid and druggable target for Chagas disease. A review published in 2015 highlights inhibitors tested against cruzain [83].

### Cryptosporidium parvum

Cryptosporidiosis, caused by *Cryptosporidium* sp., is a concern worldwide. In a large study in sub-Saharan Africa and southern Asia, this protozoan was the second most common cause of moderate-to-severe diarrhea in infants and the third most common cause in toddlers, accounting for an estimated 2.9 and 4.7 million cases annually in children <2 years old in these regions, respectively [84]. Acquired immune deficiency syndrome (AIDS) patients, people under immunosuppressive treatments, patients with inheritable immunodeficiency, diabetic people, infants, and old or malnourished people are the most susceptible to severe cryptosporidiosis [85]. Outbreaks have been described among caregivers and students of veterinary hospitals after contact with calves infected with *C*. *parvum*, the zoonotic species [86]. A major outbreak was reported in Milwaukee in 1993, in which 403,000 people were infected and the cost of outbreak-associated illness was US$96.2 million [87, 88].

Genes encoding 20 clan CA cathepsin L-like proteases have been reported in *Cryptosporidium parvum*. Five genes coding for cryptopains—cathepsin L-like proteases, a representative of clan CA—were identified in the *C*. *parvum* genome [89], but only cryptopain-1 has been analyzed biochemically [90]. Cryptopain-1 is actively transcribed and expressed in sporozoites, the infectious stage of the parasite [90]. At physiologically achievable concentrations, K11777 arrested the growth of *C*. *parvum* in human intestinal cell lines [91]. In C57BL/6 interferon-γ receptor knockout mice, which are highly susceptible to *C*. *parvum* infection (mimicking cryptosporidiosis in AIDS patients), K11777 administered either orally or intraperitoneally rescued mice from a lethal *C*. *parvum* infection [91].This potent anticryptosporidial activity is hypothesized to be the result of K11777 inhibiting the active site of cryptopain-1 [91].

Recently, otubain protease (OTU), a CP that participates in the ubiquitin pathway, has also been identified [92]. The biochemical properties of the otubain-like CP of *C*. *parvum* (CpOTU) were characterized, and the enzyme may have an essential function during the oocyst stage of the parasite, when its expression reached maximum levels [93]. The protein contains an unusual C-terminal extension (217 amino acids) compared to other OTUs previously identified in human, mouse, and *Drosophila*, and deletion of the extension resulted in complete loss of enzyme activity.

### *Plasmodium* sp.

Malaria is by far the deadliest parasitic disease of humans, with 446,000 or 631,000 [94, **95**] deaths estimated in 2015 using different modeling approaches. *Plasmodium falciparum* and *P*. *vivax* are the most common species infecting humans, with *P*. *falciparum* responsible for nearly all deaths. The parasites express multiple CPs, some of which are the subjects of recent reviews [96–98]. For *P*. *falciparum*, the genome sequence predicts 33 CP-like proteins, although a number of these are probably not active enzymes. The best characterized are 4 falcipains and 3 dipeptidyl peptidases, all clan CA proteases [96].

The functions of plasmodial CPs have been characterized using selective CP inhibitors and in some cases by gene knockout. Erythrocytic malaria parasites import erythrocyte cytosol and degrade hemoglobin in an acidic food vacuole as a source of amino acids **[99]** in a cooperative process involving enzymes of multiple catalytic classes, including CPs, aspartic proteases, and metalloproteases. Incubating parasites with broadly active CP inhibitors causes the food vacuole to swell and fill with undegraded hemoglobin, suggesting CP essentiality in hemoglobin processing. CPs also contribute indirectly to hemoglobin hydrolysis via the processing of plasmepsins to active enzymes [100]. The CPs with clear roles in hemoglobin hydrolysis are falcipain-2, falcipain-3, and dipeptidyl aminopeptidase 1. Knockout of falcipain-2 caused the food vacuoles of *P*. *falciparum* trophozoites to fill with undegraded hemoglobin, but this abnormality resolved later in the life cycle, presumably due to expression of falcipain-3 [101]. In contrast, knockout of falcipain-3 was not tolerated, suggesting that this protease is essential [102]. Some studies have also suggested roles for CPs in erythrocyte rupture at the completion of the erythrocytic cycle or in merozoite invasion of erythrocytes [103]. CP inhibitors blocked the rupture of erythrocytes by mature schizonts. Mediators of this process are predicted to be members of the SERA family, including the pseudo-CP SERA5 **[104]**, the functional CP SERA6 [105], and dipeptidyl aminopeptidase 3 [103, 106], although recent reports refute activity of dipeptidyl aminopeptidase 3 in erythrocyte rupture [98, 107]. Recent advances have demonstrated a proteolytic cascade responsible for egress of merozoites from host erythrocytes; this cascade includes cleavage of the actin-binding domain of the erythrocyte cytoskeletal protein β-spectrin by SERA6 to mediate erythrocyte rupture, the final step required for merozoite egress [108]. Some studies have also suggested that CPs participate in erythrocyte invasion by asexual parasites, notably falcipain-1 **[109]** and dipeptidyl aminopeptidase 3 [98]. However, studies with protease inhibitors have generally not supported this conclusion; knockout of falcipain-1 did not block *P*. *falciparum* development **[110, 111]**, and antibodies against the endogenous *P*. *falciparum* CP inhibitor falstatin blocked invasion, suggesting that inhibiting falcipain-like CP activity facilitates invasion [112]. Considering another family of clan CA CP, a nucleolar calpain-like *P*. *falciparum* CP appears to be required for the development of erythrocytic parasites [113, 114]. Also, a *P*. *falciparum* otubain-like CP was recently shown to localize to the apicoplast organelle and to be required for normal apicoplast and parasite development via inhibition of the predicted role of *P*. *falciparum* Atg8 in protein import to the apicoplast [115].

CPs appear to play additional roles in nonerythrocytic plasmodial stages. Considering liver stages, an unidentified plasmodial CP cleaves the circumsporozoite protein, which coats the sporozoites injected by mosquitoes, to enable invasion of hepatocytes [116]. In the murine parasite *P*. *berghei*, the orthologue of falcipain-1 appears to be critical for invasion of erythrocytes by hepatocyte-derived merozoites [117]. Considering mosquito stages, CP inhibitors and the knockout of falcipain-1 decreased oocyst production in mosquitoes [110, 118]. Also, dipeptidyl aminopeptidase 2, which is expressed in gametocytes, may contribute to gamete egress **[119]**.

Our understanding of the roles of CPs in the plasmodial life cycle suggests numerous potential drug targets. Falcipain-2 and falcipain-3 have low pH optima, consistent with activity in the acidic food vacuole, and both enzymes were localized to the food vacuole [120]. Falcipain-2 is more active against peptidyl substrates, uniquely able to activate and undergo autohydrolysis at neutral pH, and more stable at neutral pH. Considering specificity for peptide substrates and inhibitors, important differences were seen between falcipain-2, falcipain-3, and homologs from the rodent parasites *P*. *berghei* and *P*. *vinckei*; differences in specificity between falcipain-2 and falcipain-3 were less pronounced [121]. Both enzymes are synthesized as membrane-bound proforms that are processed, probably by autohydrolysis, to soluble mature forms. A related enzyme, falcipain-2′, is nearly identical in sequence and biochemical features to falcipain-2, but its role is uncertain, as in contrast to the case with falcipain-2 and falcipain-3, knockout of falcipain-2′ had no clear phenotype [122]. The falcipains have some unique features for papain-family proteases, including unusually long N-terminal domains and insertions in the catalytic domain. Identified functions of these domains include trafficking of falcipain-2 to the food vacuole by upstream portions of the prodomain; enzyme inhibition by downstream portions of the prodomain; mediation of enzyme folding by short peptides immediately upstream of the catalytic domain **[123]**; and mediation of binding to the native substrate, hemoglobin, by a small insertion near the C-terminus of the catalytic domain.

Multiple studies have demonstrated that CP inhibitors have potent antimalarial effects [124]. With these inhibitors, a block in *P*. *falciparum* development is accompanied by a specific block in hemoglobin hydrolysis, marked by the appearance of swollen, hemoglobin-filled food vacuoles, and antiparasitic effects correlated with the degree of inhibition of falcipain-2 and falcipain-3. Drug discovery directed against falcipains is facilitated by the available structures of falcipain-2 and falcipain-3 complexed with small-molecule and protein inhibitors **[125]**.

Peptidyl falcipain inhibitors with nanomolar antimalarial activity have included fluoromethyl ketones, vinyl sulfones, and aldehydes; in some cases, in vivo activity against murine malaria has also been demonstrated [126]. Promising nonpeptidyl falcipain inhibitors have included a series of nitriles that was extensively studied with many promising features, including excellent in vitro and in vivo potency [127]; this project was halted because of tissue binding that might predict idiosyncratic toxicity, but the evaluation of nitrile inhibitors is ongoing. Another interesting approach is the optimization of natural products, including analogues of gallinamide A, a compound from cyanobacteria with nanomolar antimalarial activity [128], and sugarcane cystatin [129].

Concerning the potential for resistance, parasites were selected for resistance to a vinyl sulfone falcipain inhibitor, but the selection was slow and the mechanism of resistance complex, without mutations in target enzymes, suggesting that resistance to falcipain inhibitors may develop slowly, especially with combination therapy [130]. Considering combinations, the activity of artemisinins, the mainstay of modern treatment for falciparum malaria, requires falcipain activity **[131]**; thus, falcipain inhibitors should probably not be combined with artemisinins. In contrast, inhibitors of CPs and aspartic proteases showed synergistic antimalarial effects, consistent with a complementary role for these two classes of enzymes and suggesting the potential for synergistic combination antimalarial therapy [132].

### Toxoplasma gondii

*Toxoplasma gondii* is a foodborne pathogen with seroprevalence ranging from 10%–30% in North America and northern Europe to more than 80% in areas of Latin America and Africa [133]. Most infected individuals remain asymptomatic despite lifelong infection. In contrast, congenital transmission or infection of immunocompromised patients with AIDS or organ transplants can lead to fatal, disseminated disease [133]. *T*. *gondii* CPs have been shown to be important for invasion, digestion of host proteins for nutrition [134, 137], and autophagy for cyst survival [138]. The *Toxoplasma* genome project revealed that the redundancy of CP genes is lower in *T*. *gondii* than in most other studied parasites. For example, *E*. *histolytica* has more than 50 genes encoding CPs with similar structure and specificity [139]. In contrast, *T*. *gondii* has genes encoding only one cathepsin B (TgCPB), one cathepsin L (TgCPL), and three cathepsin Cs (TgCPC1, 2, and 3), potentially making them more amenable drug targets. Active recombinant proteases have been expressed for all the *T*. *gondii* CPs, simplifying structure-based drug design.

TgCPB and TgCPL have been linked to host cell invasion. Targeting TgCPB with a peptidyl cathepsin B inhibitor or antisense RNA inhibited host cell invasion and in vitro growth and blocked infection in a chick embryo model of toxoplasmosis [142]. TgCPL acts as a maturase for TgCPB **[143]** and key adhesins, the microneme proteins MIC2-associated protein (M2AP) and MIC3. TgCPL knockouts were attenuated in virulence in acute infection in mice [134]. Both TgCPB and TgCPL have been localized in the vacuolar compartment, a lysosome-like organelle, where TgCPL has been shown to digest host cytosolic proteins. Most recently, TgCPL has been shown to be important for cyst survival in latent infection [138]. In TgCPL knockout strains or cysts incubated with the vinyl sulfone inhibitor LHVS, autophagy of autophagosomes in the vacuolar compartment was inhibited, resulting in abnormal cyst morphology and decreased survival.

The most developed peptidyl inhibitors target TgCPB and/or TgCPL. The crystal structure of TgCPL with morpholinurea-leucyl-homophenyl-vinyl sulfone has been determined, and the inhibitor can block host cell invasion [136] and cyst viability in vitro [138]. K11777 inhibits purified recombinant TgCPB and TgCPL in the nanomolar range and blocks host cell invasion, parasite replication, and viability in a chick embryo egg model [144]. Unfortunately, neither vinyl sulfone inhibitor is likely to cross the blood–brain barrier to prevent latent infection, so further optimization will be required.

The *T*. *gondii* cathepsin Cs are exopeptidases that are also potential drug targets. TgCPC1 was the most highly expressed cathepsin mRNA in tachyzoites; TgCPC3 was only identified in oocysts [137]. Both TgCPC1 and TgCPC2 localize to the dense granules and are secreted into the parasitophorous vacuole, where they degrade peptides. Both TgCPC1 and TgCPC2 were inhibited by Gly-Phe-dimethylketone, reducing parasite intracellular growth and proliferation. The same phenotype was not seen with a TgCPC1 knockout, as TgCPC2 expression was up-regulated, suggesting the importance of inhibiting both enzymes [137].

Autophagy is a key process in all eukaryotic cells to remove and recycle misfolded proteins and damaged organelles. Autophagy is likely to be important in *T*. *gondii* tachyzoites to survive extracellular stress and for bradyzoites during latent infection [138]. Although only a limited number of autophagy proteins (Atg) are encoded in the *T*. *gondii* genome, and there are no classic lysosomes in this organism, autophagosome-like bodies are formed [138, 145]. TgCPL appears to play an important role in the degradation of autophagosomes, as knockout or inhibition of TgCPL results in undigested proteins and organelles in the vacuolar compartment, limiting chronic infection in mice.

The clan CA cysteine proteinases of *T*. *gondii* have been identified as potential vaccine candidates. DNA vaccines containing TgCPB or TgCPL gene sequences individually or together produced both humoral and cellular immune responses in BALB/c mice. Following immunization, survival was prolonged following intraperitoneal challenge with tachyzoites, with the most significant effect from the combined TgCPB/TgCPL vaccine [146]. Similar results were seen with a TgCPC1 DNA vaccine [147].

## Conclusion

From the 7 protozoan parasites causing human disease that are of interest in this review, only 2 genera (*Trypanosoma* and *Plasmodium*) have at least one well-characterized clan CA CP: cruzain in *T*. *cruzi*, TbCatL in *T*. *brucei*, and falcipain-2 and falcipain-3 in the malaria parasite. These enzymes were validated as drug targets using a variety of inhibitor chemistries.

One common theme is the observation that distinct pathogens employ related CPs to perform similar functions. For example, the process of invading a tissue in the case of extracellular parasites [10, 19, 51, 148] and invading a host cell in the case of intracellular parasites [71, 135, 136, 140] is highly dependent on clan CA proteases. It is also interesting to note that parasites have evolved different mechanisms to utilize CPs to modulate the immune system of the host. EhCP can directly degrade IgA, IgG, and IL-18 [11–15]. By contrast, CPB in *Leishmania* sp. modulates host responses by down-regulating protective Th1 immune responses, and in particular IFN-γ production, via degradation of the transcription factor NF-κB and the subsequent inhibition of IL-12 production by infected macrophages [26, 35, 149].

Apart from the more well-studied *Trypanosoma* and *Plasmodium* sp., it is important to continue investigations regarding the therapeutic potential of other protozoan CPs. In support of this, essentiality has been suggested or demonstrated for the CPs in many of the species discussed here. Furthermore, the potential for the emergence of other protease targets is great, considering that less than 10% of putative CPs found in the respective genomes have been so far characterized.

**Key learning points**

- Gaps in our knowledge: there are many genome copies of CPs per protozoan organism. Less than 10 enzymes have been well characterized per parasite and not more than 2 enzymes per pathogen have had their structures resolved by X-ray crystallography.
- Importance: the major functions of CPs shared by these protozoan parasites are host invasion and tissue penetration, virulence and evasion/modulation of the host immune system.
- Potential targets for chemotherapy: the most well studied CPs of protozoan parasites are the cathepsin L-like enzymes from *T*. *brucei* and *T*. *cruzi*, and falcipain-2 and falcipain-3 from *Plasmodium* sp. They are validated targets in vitro and in vivo for the development of novel therapies.

**Top four papers**

1. McKerrow JH. Development of cysteine protease inhibitors as chemotherapy for parasitic diseases: insights on safety, target validation, and mechanism of action. Int J Parasitol. 1999;29(6):833–7. PubMed PMID: 10480720. **[162]**
2. Sijwali PS, Rosenthal PJ. Gene disruption confirms a critical role for the cysteine protease falcipain-2 in hemoglobin hydrolysis by Plasmodium falciparum. Proc Natl Acad Sci U S A. 2004;101(13):4384–9. PubMed PMID: 15070727.
3. Sajid M, McKerrow JH. Cysteine proteases of parasitic organisms. Mol Biochem Parasitol. 2002;120(1):1–21. PubMed PMID: 11849701.
4. McKerrow JH, Caffrey C, Kelly B, Loke P, Sajid M. Proteases in parasitic diseases. Annu Rev Pathol. 2006;1:497–536. doi: 10.1146/annurev.pathol.1.110304.100151. PubMed PMID: 18039124 **[163]**

## References

[1] Sajid M, McKerrow JH. Cysteine proteases of parasitic organisms. Mol Biochem Parasitol. 2002;120(1):1–21. . 11849701 10.1016/s0166-6851(01)00438-8

[2] Barr SC, Warner KL, Kornreic BG, Piscitelli J, Wolfe A, Benet L, et al A cysteine protease inhibitor protects dogs from cardiac damage during infection by Trypanosoma cruzi. Antimicrob Agents Chemother. 2005;49(12):5160–1. 10.1128/AAC.49.12.5160-5161.2005 ; PubMed Central PMCID: PMCPMC1315979. 16304193 PMC1315979

[3] Engel JC, Doyle PS, Hsieh I, McKerrow JH. Cysteine protease inhibitors cure an experimental Trypanosoma cruzi infection. J Exp Med. 1998;188(4):725–34. ; PubMed Central PMCID: PMCPMC2213346. 9705954 10.1084/jem.188.4.725 PMC2213346

[4] Lozano R, Naghavi M, Foreman K, Lim S, Shibuya K, Aboyans V, et al Global and regional mortality from 235 causes of death for 20 age groups in 1990 and 2010: a systematic analysis for the Global Burden of Disease Study 2010. Lancet. 2012;380(9859):2095–128. 10.1016/S0140-6736(12)61728-0 . 23245604 PMC10790329

[5] Nakajima H. The world health report 1998—Life in the 21st century: A vision for all World Health Organization, 1998.

[6] Reed SL, Keene WE, McKerrow JH. Thiol proteinase expression and pathogenicity of Entamoeba histolytica. J Clin Microbiol. 1989;27(12):2772–7. ; PubMed Central PMCID: PMCPMC267124. 2556432 10.1128/jcm.27.12.2772-2777.1989 PMC267124

[7] Moncada D, Keller K, Chadee K. Entamoeba histolytica cysteine proteinases disrupt the polymeric structure of colonic mucin and alter its protective function. Infect Immun. 2003;71(2):838–44. 10.1128/IAI.71.2.838-844.2003 ; PubMed Central PMCID: PMCPMC145371. 12540564 PMC145371

[8] Lauwaet T, Oliveira MJ, Callewaert B, De Bruyne G, Saelens X, Ankri S, et al Proteolysis of enteric cell villin by Entamoeba histolytica cysteine proteinases. J Biol Chem. 2003;278(25):22650–6. 10.1074/jbc.M300142200 . 12690119

[9] Espinosa-Cantellano M, Martinez-Palomo A. Pathogenesis of intestinal amebiasis: from molecules to disease. Clin Microbiol Rev. 2000;13(2):318–31. ; PubMed Central PMCID: PMCPMC100155. 10756002 10.1128/cmr.13.2.318-331.2000 PMC100155

[10] Debnath A, Tashker JS, Sajid M, McKerrow JH. Transcriptional and secretory responses of Entamoeba histolytica to mucins, epithelial cells and bacteria. Int J Parasitol. 2007;37(8–9):897–906. 10.1016/j.ijpara.2007.01.016 . 17362964

[11] Garcia-Nieto RM, Rico-Mata R, Arias-Negrete S, Avila EE. Degradation of human secretory IgA1 and IgA2 by Entamoeba histolytica surface-associated proteolytic activity. Parasitol Int. 2008;57(4):417–23. 10.1016/j.parint.2008.04.013 . 18571975

[12] Tran VQ, Herdman DS, Torian BE, Reed SL. The neutral cysteine proteinase of Entamoeba histolytica degrades IgG and prevents its binding. J Infect Dis. 1998;177(2):508–11. . 9466550 10.1086/517388

[13] Reed SL, Ember JA, Herdman DS, DiScipio RG, Hugli TE, Gigli I. The extracellular neutral cysteine proteinase of Entamoeba histolytica degrades anaphylatoxins C3a and C5a. J Immunol. 1995;155(1):266–74. . 7602103

[14] Zhang Z, Wang L, Seydel KB, Li E, Ankri S, Mirelman D, et al Entamoeba histolytica cysteine proteinases with interleukin-1 beta converting enzyme (ICE) activity cause intestinal inflammation and tissue damage in amoebiasis. Mol Microbiol. 2000;37(3):542–8. . 10931347 10.1046/j.1365-2958.2000.02037.x

[15] Que X, Kim SH, Sajid M, Eckmann L, Dinarello CA, McKerrow JH, et al A surface amebic cysteine proteinase inactivates interleukin-18. Infect Immun. 2003;71(3):1274–80. 10.1128/IAI.71.3.1274-1280.2003 ; PubMed Central PMCID: PMCPMC148873. 12595442 PMC148873

[16] St-Pierre J, Moreau F, Cornick S, Quach J, Begum S, Aracely Fernandez L, et al The macrophage cytoskeleton acts as a contact sensor upon interaction with Entamoeba histolytica to trigger IL-1beta secretion. PLoS Pathog. 2017;13(8):e1006592 10.1371/journal.ppat.1006592 ; PubMed Central PMCID: PMCPMC5587335. 28837696 PMC5587335

[17] Ebert F, Bachmann A, Nakada-Tsukui K, Hennings I, Drescher B, Nozaki T, et al An Entamoeba cysteine peptidase specifically expressed during encystation. Parasitol Int. 2008;57(4):521–4. 10.1016/j.parint.2008.07.002 . 18723116

[18] Irmer H, Tillack M, Biller L, Handal G, Leippe M, Roeder T, et al Major cysteine peptidases of Entamoeba histolytica are required for aggregation and digestion of erythrocytes but are dispensable for phagocytosis and cytopathogenicity. Mol Microbiol. 2009;72(3):658–67. 10.1111/j.1365-2958.2009.06672.x . 19426210

[19] Melendez-Lopez SG, Herdman S, Hirata K, Choi MH, Choe Y, Craik C, et al Use of recombinant Entamoeba histolytica cysteine proteinase 1 to identify a potent inhibitor of amebic invasion in a human colonic model. Eukaryot Cell. 2007;6(7):1130–6. Epub 2007/05/22. 10.1128/EC.00094-07 ; PubMed Central PMCID: PMCPmc1951106. 17513563 PMC1951106

[20] Mortality GBD, Causes of Death C. Global, regional, and national age-sex specific all-cause and cause-specific mortality for 240 causes of death, 1990–2013: a systematic analysis for the Global Burden of Disease Study 2013. Lancet. 2015;385(9963):117–71. 10.1016/S0140-6736(14)61682-2 ; PubMed Central PMCID: PMCPMC4340604. 25530442 PMC4340604

[21] McCall LI, Zhang WW, Matlashewski G. Determinants for the development of visceral leishmaniasis disease. PLoS Pathog. 2013;9(1):e1003053 10.1371/journal.ppat.1003053 ; PubMed Central PMCID: PMCPMC3536654. 23300451 PMC3536654

[22] Alexander J, Coombs GH, Mottram JC. Leishmania mexicana cysteine proteinase-deficient mutants have attenuated virulence for mice and potentiate a Th1 response. J Immunol. 1998;161(12):6794–801. Epub 1998/12/23. . 9862710

[23] Novinec M, Lenarcic B. Papain-like peptidases: structure, function, and evolution. Biomol Concepts. 2013;4(3):287–308. 10.1515/bmc-2012-0054 . 25436581

[24] Turk V, Stoka V, Vasiljeva O, Renko M, Sun T, Turk B, et al Cysteine cathepsins: from structure, function and regulation to new frontiers. Biochim Biophys Acta. 2012;1824(1):68–88. 10.1016/j.bbapap.2011.10.002 . 22024571 PMC7105208

[25] Souza AE, Waugh S, Coombs GH, Mottram JC. Characterization of a multi-copy gene for a major stage-specific cysteine proteinase of Leishmania mexicana. FEBS Lett. 1992;311(2):124–7. . 1397299 10.1016/0014-5793(92)81382-v

[26] Mottram JC, Coombs GH, Alexander J. Cysteine peptidases as virulence factors of Leishmania. Curr Opin Microbiol. 2004;7(4):375–81. ISI:000223458200010. 10.1016/j.mib.2004.06.010 15358255

[27] Mundodi V, Somanna A, Farrell PJ, Gedamu L. Genomic organization and functional expression of differentially regulated cysteine protease genes of Leishmania donovani complex. Gene. 2002;282(1–2):257–65. . 11814698 10.1016/s0378-1119(01)00851-4

[28] Bart G, Coombs GH, Mottram JC. Isolation of lmcpc, a gene encoding a Leishmania mexicana cathepsin-B-like cysteine proteinase. Mol Biochem Parasitol. 1995;73(1–2):271–4. . 8577339 10.1016/0166-6851(95)00113-f

[29] Mottram JC, Robertson CD, Coombs GH, Barry JD. A developmentally regulated cysteine proteinase gene of Leishmania mexicana. Mol Microbiol. 1992;6(14):1925–32. . 1508041 10.1111/j.1365-2958.1992.tb01365.x

[30] Mottram JC, Frame MJ, Brooks DR, Tetley L, Hutchison JE, Souza AE, et al The multiple cpb cysteine proteinase genes of Leishmania mexicana encode isoenzymes that differ in their stage regulation and substrate preferences. J Biol Chem. 1997;272(22):14285–93. 10.1074/jbc.272.22.14285. WOS:A1997XB49200050. 9162063

[31] Mundodi V, Kucknoor AS, Gedamu L. Role of Leishmania (Leishmania) chagasi amastigote cysteine protease in intracellular parasite survival: studies by gene disruption and antisense mRNA inhibition. BMC Mol Biol. 2005;6:3 10.1186/1471-2199-6-3. PMC549197. 15691375 PMC549197

[32] Denise H, Poot J, Jiménez M, Ambit A, Herrmann DC, Vermeulen AN, et al Studies on the CPA cysteine peptidase in the Leishmania infantum genome strain JPCM5. BMC Mol Biol. 2006;7:42 10.1186/1471-2199-7-42. PMC1657026. 17101050 PMC1657026

[33] Mottram JC, Souza AE, Hutchison JE, Carter R, Frame MJ, Coombs GH. Evidence from disruption of the lmcpb gene array of Leishmania mexicana that cysteine proteinases are virulence factors. P Natl Acad Sci USA. 1996;93(12):6008–13. 10.1073/pnas.93.12.6008. WOS:A1996UQ45500062. PMC39179 8650210

[34] Denise H, McNeil K, Brooks DR, Alexander J, Coombs GH, Mottram JC. Expression of multiple CPB genes encoding cysteine proteases is required for Leishmania mexicana virulence in vivo. Infect Immun. 2003;71(6):3190–5. 10.1128/IAI.71.6.3190-3195.2003. WOS:000183116300026. 12761098 PMC155739

[35] Cameron P, McGachy A, Anderson M, Paul A, Coombs GH, Mottram JC, et al Inhibition of lipopolysaccharide-induced macrophage IL-12 production by Leishmania mexicana amastigotes: the role of cysteine peptidases and the NF-kappaB signaling pathway. J Immunol. 2004;173(5):3297–304. . 15322192 10.4049/jimmunol.173.5.3297

[36] Leao SD, Lang T, Prina E, Hellio R, Antoine JC. Intracellular Leishmania-Amazonensis Amastigotes Internalize and Degrade Mhc Class-Ii Molecules of Their Host-Cells. J Cell Sci. 1995;108:3219–31. WOS:A1995RZ34900008. 7593283 10.1242/jcs.108.10.3219

[37] Casgrain PA, Martel C, McMaster WR, Mottram JC, Olivier M, Descoteaux A. Cysteine Peptidase B Regulates Leishmania mexicana Virulence through the Modulation of GP63 Expression. PLoS Pathog. 2016;12(5):e1005658 10.1371/journal.ppat.1005658 ; PubMed Central PMCID: PMCPMC4871588. 27191844 PMC4871588

[38] El-Fadili AK, Zangger H, Desponds C, Gonzalez IJ, Zalila H, Schaff C, et al Cathepsin B-like and cell death in the unicellular human pathogen Leishmania. Cell Death Dis. 2010;1:e71 Epub 2011/03/03. 10.1038/cddis.2010.51 ; PubMed Central PMCID: PMCPmc3032344. 21364675 PMC3032344

[39] Gerbaba TK, Gedamu L. Cathepsin B gene disruption induced Leishmania donovani proteome remodeling implies cathepsin B role in secretome regulation. PLoS ONE. 2013;8(11):e79951 10.1371/journal.pone.0079951 ; PubMed Central PMCID: PMCPMC3828211. 24244582 PMC3828211

[40] Bart G, Frame MJ, Carter R, Coombs GH, Mottram JC. Cathepsin B-like cysteine proteinase-deficient mutants of Leishmania mexicana. Mol Biochem Parasitol. 1997;88(1–2):53–61. Epub 1997/09/01. . 9274867 10.1016/s0166-6851(97)00072-8

[41] Williams RA, Mottram JC, Coombs GH. Distinct roles in autophagy and importance in infectivity of the two ATG4 cysteine peptidases of Leishmania major. J Biol Chem. 2013;288(5):3678–90. 10.1074/jbc.M112.415372 ; PubMed Central PMCID: PMCPMC3561585. 23166325 PMC3561585

[42] Azevedo CS, Guido BC, Pereira JL, Nolasco DO, Correa R, Magalhaes KG, et al Revealing a Novel Otubain-Like Enzyme from Leishmania infantum with Deubiquitinating Activity toward K48-Linked Substrate. Front Chem. 2017;5:13 10.3389/fchem.2017.00013 ; PubMed Central PMCID: PMCPMC5362604. 28386537 PMC5362604

[43] Selzer PM, Pingel S, Hsieh I, Ugele B, Chan VJ, Engel JC, et al Cysteine protease inhibitors as chemotherapy: lessons from a parasite target. Proc Natl Acad Sci U S A. 1999;96(20):11015–22. ; PubMed Central PMCID: PMCPMC34234. 10500116 10.1073/pnas.96.20.11015 PMC34234

[44] Paladi Cde S, Pimentel IA, Katz S, Cunha RL, Judice WA, Caires AC, et al In vitro and in vivo activity of a palladacycle complex on Leishmania (Leishmania) amazonensis. PLoS Negl Trop Dis. 2012;6(5):e1626 10.1371/journal.pntd.0001626 ; PubMed Central PMCID: PMCPMC3352823. 22616018 PMC3352823

[45] Lima CB, Arrais-Silva WW, Cunha RL, Giorgio S. A novel organotellurium compound (RT-01) as a new antileishmanial agent. Korean J Parasitol. 2009;47(3):213–8. 10.3347/kjp.2009.47.3.213 ; PubMed Central PMCID: PMCPMC2735685. 19724693 PMC2735685

[46] Salerno Pimentel IA, Paladi Cde S, Katz S, de Souza Judice WA, Cunha RL, Barbieri CL. In vitro and in vivo activity of an organic tellurium compound on Leishmania (Leishmania) chagasi. PLoS ONE. 2012;7(11):e48780 10.1371/journal.pone.0048780 ; PubMed Central PMCID: PMCPMC3492430. 23144968 PMC3492430

[47] Schroder J, Noack S, Marhofer RJ, Mottram JC, Coombs GH, Selzer PM. Identification of semicarbazones, thiosemicarbazones and triazine nitriles as inhibitors of Leishmania mexicana cysteine protease CPB. PLoS ONE. 2013;8(10):e77460 10.1371/journal.pone.0077460 ; PubMed Central PMCID: PMCPMC3797739. 24146999 PMC3797739

[48] de Almeida L, Alves KF, Maciel-Rezende CM, Jesus Lde O, Pires FR, Junior CV, et al Benzophenone derivatives as cysteine protease inhibitors and biological activity against Leishmania(L.) amazonensis amastigotes. Biomed Pharmacother. 2015;75:93–9. 10.1016/j.biopha.2015.08.030 . 26463637

[49] Caffrey CR, Lima AP, Steverding D. Cysteine peptidases of kinetoplastid parasites. Adv Exp Med Biol. 2011;712:84–99. 10.1007/978-1-4419-8414-2_6 . 21660660

[50] Grab DJ, Garcia-Garcia JC, Nikolskaia OV, Kim YV, Brown A, Pardo CA, et al Protease Activated Receptor Signaling Is Required for African Trypanosome Traversal of Human Brain Microvascular Endothelial Cells. PloS Negl Trop Dis. 2009;3(7):e479 ARTN e479 10.1371/journal.pntd.0000479. WOS:000268452200010. 19621073 PMC2707606

[51] Nikolskaia OV, de ALAP, Kim YV, Lonsdale-Eccles JD, Fukuma T, Scharfstein J, et al Blood-brain barrier traversal by African trypanosomes requires calcium signaling induced by parasite cysteine protease. J Clin Invest. 2006;116(10):2739–47. 10.1172/JCI27798 ; PubMed Central PMCID: PMCPMC1570376. 16998589 PMC1570376

[52] Alsford S, Currier RB, Guerra-Assuncao JA, Clark TG, Horn D. Cathepsin-L Can Resist Lysis by Human Serum in Trypanosoma brucei brucei. PLoS Pathog. 2014;10(5):e1004130 ARTN e1004130 10.1371/journal.ppat.1004130. WOS:000337732300038. 24830321 PMC4022737

[53] Uzureau P, Uzureau S, Lecordier L, Fontaine F, Tebabi P, Homble F, et al Mechanism of Trypanosoma brucei gambiense resistance to human serum. Nature. 2013;501(7467):430–4. 10.1038/nature12516 . 23965626

[54] Santos CC, Coombs GH, Lima AP, Mottram JC. Role of the Trypanosoma brucei natural cysteine peptidase inhibitor ICP in differentiation and virulence. Mol Microbiol. 2007;66(4):991–1002. 10.1111/j.1365-2958.2007.05970.x ; PubMed Central PMCID: PMCPMC2680270. 17944830 PMC2680270

[55] Caffrey CR, Hansell E, Lucas KD, Brinen LS, Alvarez Hernandez A, Cheng J, et al Active site mapping, biochemical properties and subcellular localization of rhodesain, the major cysteine protease of Trypanosoma brucei rhodesiense. Mol Biochem Parasitol. 2001;118(1):61–73. . 11704274 10.1016/s0166-6851(01)00368-1

[56] Mackey ZB, O'Brien, Greenbaum DC, Blank RB, McKerrow JH. A cathepsin B-like protease is required for host protein degradation in Trypanosoma brucei. J Biol Chem. 2004;279(46):48426–33. 10.1074/jbc.M402470200 . 15326171

[57] O'Brien TC, Mackey ZB, Fetter RD, Choe Y, O'Donoghue AJ, Zhou M, et al A Parasite Cysteine Protease Is Key to Host Protein Degradation and Iron Acquisition. J Biol Chem. 2008;283(43):28934–43. ISI:000260179900019. 10.1074/jbc.M805824200 18701454 PMC2570886

[58] O'Brien TC, Mackey ZB, Fetter RD, Choe Y, O'Donoghue AJ, Zhou M, et al A parasite cysteine protease is key to host protein degradation and iron acquisition. J Biol Chem. 2008;283(43):28934–43. 10.1074/jbc.M805824200 ; PubMed Central PMCID: PMCPMC2570886. 18701454 PMC2570886

[59] Steverding D, Sexton DW, Wang X, Gehrke SS, Wagner GK, Caffrey CR. Trypanosoma brucei: chemical evidence that cathepsin L is essential for survival and a relevant drug target. Int J Parasitol. 2012;42(5):481–8. 10.1016/j.ijpara.2012.03.009 . 22549023

[60] Caffrey CR, Scory S, Steverding D. Cysteine proteinases of trypanosome parasites: novel targets for chemotherapy. Curr Drug Targets. 2000;1(2):155–62. . 11465068 10.2174/1389450003349290

[61] Scory S, Caffrey CR, Stierhof YD, Ruppel A, Steverding D. Trypanosoma brucei: killing of bloodstream forms in vitro and in vivo by the cysteine proteinase inhibitor Z-phe-ala-CHN2. Exp Parasitol. 1999;91(4):327–33. 10.1006/expr.1998.4381 . 10092476

[62] Steverding D. Evaluation of trypanocidal activity of combinations of anti-sleeping sickness drugs with cysteine protease inhibitors. Exp Parasitol. 2015;151–152:28–33. 10.1016/j.exppara.2015.01.016 . 25662707

[63] Schirmeister T, Schmitz J, Jung S, Schmenger T, Krauth-Siegel RL, Gutschow M. Evaluation of dipeptide nitriles as inhibitors of rhodesain, a major cysteine protease of Trypanosoma brucei. Bioorg Med Chem Lett. 2017;27(1):45–50. 10.1016/j.bmcl.2016.11.036 . 27890381

[64] Ehmke V, Winkler E, Banner DW, Haap W, Schweizer WB, Rottmann M, et al Optimization of triazine nitriles as rhodesain inhibitors: structure-activity relationships, bioisosteric imidazopyridine nitriles, and X-ray crystal structure analysis with human cathepsin L. ChemMedChem. 2013;8(6):967–75. 10.1002/cmdc.201300112 . 23658062

[65] Ferreira LG, Andricopulo AD. Targeting cysteine proteases in trypanosomatid disease drug discovery. Pharmacol Ther. 2017;180:49–61. 10.1016/j.pharmthera.2017.06.004 . 28579388

[66] Perez-Molina JA, Molina I. Chagas disease. Lancet. 2017;391(10115):82–94. 10.1016/S0140-6736(17)31612-4 . 28673423

[67] Scharfstein J, Schechter M, Senna M, Peralta JM, Mendonca-Previato L, Miles MA. Trypanosoma cruzi: characterization and isolation of a 57/51,000 m.w. surface glycoprotein (GP57/51) expressed by epimastigotes and bloodstream trypomastigotes. J Immunol. 1986;137(4):1336–41. . 3090146

[68] Duschak VG, Couto AS. Cruzipain, the major cysteine protease of Trypanosoma cruzi: a sulfated glycoprotein antigen as relevant candidate for vaccine development and drug target. A review. Curr Med Chem. 2009;16(24):3174–202. . 19689291 10.2174/092986709788802971

[69] Franke de Cazzulo BM, Martinez J, North MJ, Coombs GH, Cazzulo JJ. Effects of proteinase inhibitors on the growth and differentiation of Trypanosoma cruzi. FEMS Microbiol Lett. 1994;124(1):81–6. . 8001773 10.1111/j.1574-6968.1994.tb07265.x

[70] Cazzulo JJ, Stoka V, Turk V. Cruzipain, the major cysteine proteinase from the protozoan parasite Trypanosoma cruzi. Biol Chem. 1997;378(1):1–10. . 9049059 10.1515/bchm.1997.378.1.1

[71] Meirelles MN, Juliano L, Carmona E, Silva SG, Costa EM, Murta AC, et al Inhibitors of the major cysteinyl proteinase (GP57/51) impair host cell invasion and arrest the intracellular development of Trypanosoma cruzi in vitro. Mol Biochem Parasitol. 1992;52(2):175–84. . 1620157 10.1016/0166-6851(92)90050-t

[72] Eakin AE, Mills AA, Harth G, McKerrow JH, Craik CS. The sequence, organization, and expression of the major cysteine protease (cruzain) from Trypanosoma cruzi. J Biol Chem. 1992;267(11):7411–20. . 1559982

[73] McGrath ME, Eakin AE, Engel JC, McKerrow JH, Craik CS, Fletterick RJ. The crystal structure of cruzain: a therapeutic target for Chagas' disease. J Mol Biol. 1995;247(2):251–9. 10.1006/jmbi.1994.0137 . 7707373

[74] Yong V, Schmitz V, Vannier-Santos MA, de Lima AP, Lalmanach G, Juliano L, et al Altered expression of cruzipain and a cathepsin B-like target in a Trypanosoma cruzi cell line displaying resistance to synthetic inhibitors of cysteine-proteinases. Mol Biochem Parasitol. 2000;109(1):47–59. . 10924756 10.1016/s0166-6851(00)00237-1

[75] Kosec G, Alvarez V, Cazzulo JJ. Cysteine proteinases of Trypanosoma cruzi: from digestive enzymes to programmed cell death mediators. Biocell. 2006;30(3):479–90. . 17375468

[76] Vanrell MC, Losinno AD, Cueto JA, Balcazar D, Fraccaroli LV, Carrillo C, et al The regulation of autophagy differentially affects Trypanosoma cruzi metacyclogenesis. PLoS Negl Trop Dis. 2017;11(11):e0006049 10.1371/journal.pntd.0006049 . 29091711 PMC5683653

[77] Ferreira RS, Bryant C, Ang KK, McKerrow JH, Shoichet BK, Renslo AR. Divergent modes of enzyme inhibition in a homologous structure-activity series. J Med Chem. 2009;52(16):5005–8. 10.1021/jm9009229 ; PubMed Central PMCID: PMCPMC3760508. 19637873 PMC3760508

[78] Fonseca NC, da Cruz LF, da Silva Villela F, do Nascimento Pereira GA, de Siqueira-Neto JL, Kellar D, et al Synthesis of a sugar-based thiosemicarbazone series and structure-activity relationship versus the parasite cysteine proteases rhodesain, cruzain, and Schistosoma mansoni cathepsin B1. Antimicrob Agents Chemother. 2015;59(5):2666–77. 10.1128/AAC.04601-14 ; PubMed Central PMCID: PMCPMC4394791. 25712353 PMC4394791

[79] Chiyanzu I, Hansell E, Gut J, Rosenthal PJ, McKerrow JH, Chibale K. Synthesis and evaluation of isatins and thiosemicarbazone derivatives against cruzain, falcipain-2 and rhodesain. Bioorg Med Chem Lett. 2003;13(20):3527–30. Epub 2003/09/25. S0960894X0300756X [pii]. . 14505663 10.1016/s0960-894x(03)00756-x

[80] Stoch SA, Zajic S, Stone JA, Miller DL, van Bortel L, Lasseter KC, et al Odanacatib, a selective cathepsin K inhibitor to treat osteoporosis: safety, tolerability, pharmacokinetics and pharmacodynamics—results from single oral dose studies in healthy volunteers. Br J Clin Pharmacol. 2013;75(5):1240–54. 10.1111/j.1365-2125.2012.04471.x ; PubMed Central PMCID: PMCPMC3635595. 23013236 PMC3635595

[81] Ndao M, Beaulieu C, Black WC, Isabel E, Vasquez-Camargo F, Nath-Chowdhury M, et al Reversible cysteine protease inhibitors show promise for a Chagas disease cure. Antimicrob Agents Chemother. 2014;58(2):1167–78. 10.1128/AAC.01855-13 ; PubMed Central PMCID: PMCPMC3910870. 24323474 PMC3910870

[82] Jones BD, Tochowicz A, Tang Y, Cameron MD, McCall LI, Hirata K, et al Synthesis and Evaluation of Oxyguanidine Analogues of the Cysteine Protease Inhibitor WRR-483 against Cruzain. ACS Med Chem Lett. 2016;7(1):77–82. 10.1021/acsmedchemlett.5b00336 ; PubMed Central PMCID: PMCPMC4716606. 26819670 PMC4716606

[83] Martinez-Mayorga K, Byler KG, Ramirez-Hernandez AI, Terrazas-Alvares DE. Cruzain inhibitors: efforts made, current leads and a structural outlook of new hits. Drug Discov Today. 2015;20(7):890–8. 10.1016/j.drudis.2015.02.004 . 25697479

[84] Sow SO, Muhsen K, Nasrin D, Blackwelder WC, Wu Y, Farag TH, et al The Burden of Cryptosporidium Diarrheal Disease among Children < 24 Months of Age in Moderate/High Mortality Regions of Sub-Saharan Africa and South Asia, Utilizing Data from the Global Enteric Multicenter Study (GEMS). PLoS Negl Trop Dis. 2016;10(5):e0004729 10.1371/journal.pntd.0004729 ; PubMed Central PMCID: PMCPMC4878811. 27219054 PMC4878811

[85] Armson A, Thompson RC, Reynoldson JA. A review of chemotherapeutic approaches to the treatment of cryptosporidiosis. Expert Rev Anti Infect Ther. 2003;1(2):297–305. . 15482125 10.1586/14787210.1.2.297

[86] Benschop J, Booker CM, Shadbolt T, Weston JF. A Retrospective Cohort Study of an Outbreak of Cryptosporidiosis among Veterinary Students. Vet Sci. 2017;4(2). 10.3390/vetsci4020029 . 29056688 PMC5606607

[87] Mac Kenzie WR, Hoxie NJ, Proctor ME, Gradus MS, Blair KA, Peterson DE, et al A massive outbreak in Milwaukee of cryptosporidium infection transmitted through the public water supply. N Engl J Med. 1994;331(3):161–7. 10.1056/NEJM199407213310304 . 7818640

[88] Corso PS, Kramer MH, Blair KA, Addiss DG, Davis JP, Haddix AC. Cost of illness in the 1993 waterborne Cryptosporidium outbreak, Milwaukee, Wisconsin. Emerg Infect Dis. 2003;9(4):426–31. 10.3201/eid0904.020417 ; PubMed Central PMCID: PMCPMC2957981. 12702221 PMC2957981

[89] Abrahamsen MS, Templeton TJ, Enomoto S, Abrahante JE, Zhu G, Lancto CA, et al Complete genome sequence of the apicomplexan, Cryptosporidium parvum. Science. 2004;304(5669):441–5. 10.1126/science.1094786 . 15044751

[90] Na BK, Kang JM, Cheun HI, Cho SH, Moon SU, Kim TS, et al Cryptopain-1, a cysteine protease of Cryptosporidium parvum, does not require the pro-domain for folding. Parasitology. 2009;136(2):149–57. 10.1017/S0031182008005350 . 19091155

[91] Ndao M, Nath-Chowdhury M, Sajid M, Marcus V, Mashiyama ST, Sakanari J, et al A cysteine protease inhibitor rescues mice from a lethal Cryptosporidium parvum infection. Antimicrob Agents Chemother. 2013;57(12):6063–73. 10.1128/AAC.00734-13 ; PubMed Central PMCID: PMCPMC3837922. 24060869 PMC3837922

[92] Nijman SM, Luna-Vargas MP, Velds A, Brummelkamp TR, Dirac AM, Sixma TK, et al A genomic and functional inventory of deubiquitinating enzymes. Cell. 2005;123(5):773–86. 10.1016/j.cell.2005.11.007 . 16325574

[93] Ju HL, Kang JM, Noh HS, Kim DR, Hong Y, Sohn WM, et al Characterization of a novel otubain-like cysteine protease of Cryptosporidium parvum. Parasitol Int. 2014;63(4):580–3. 10.1016/j.parint.2014.03.005 . 24709083

[94] Gething PW, Casey DC, Weiss DJ, Bisanzio D, Bhatt S, Cameron E, et al Mapping Plasmodium falciparum Mortality in Africa between 1990 and 2015. N Engl J Med. 2016;375(25):2435–45. 10.1056/NEJMoa1606701 ; PubMed Central PMCID: PMCPMC5484406. 27723434 PMC5484406

[95] Ghebreyesus TA. World malaria report 2017: Foreword World Health Organization, 2017 Accessed 15 Jun 2018. Available from: http://apps.who.int/iris/bitstream/handle/10665/259492/9789241565523-eng.pdf?sequence=1.

[96] Rosenthal PJ. Falcipains and other cysteine proteases of malaria parasites. Adv Exp Med Biol. 2011;712:30–48. 10.1007/978-1-4419-8414-2_3 . 21660657

[97] Rosenthal PJ. Falcipains In: Rawlings ND, Salvesen GS, editors. Handbook of Proteolytic Enzymes. Oxford: Academic Press; 2013 p. 1907–12.

[98] Deu E. Proteases as antimalarial targets: strategies for genetic, chemical, and therapeutic validation. FEBS J. 2017;284(16):2604–28. 10.1111/febs.14130 ; PubMed Central PMCID: PMCPMC5575534. 28599096 PMC5575534

[99] Francis SE, Sullivan DJ Jr., Goldberg DE. Hemoglobin metabolism in the malaria parasite Plasmodium falciparum. Annu Rev Microbiol. 1997;51:97–123. 10.1146/annurev.micro.51.1.97 . 9343345

[100] Drew ME, Banerjee R, Uffman EW, Gilbertson S, Rosenthal PJ, Goldberg DE. Plasmodium food vacuole plasmepsins are activated by falcipains. J Biol Chem. 2008;283(19):12870–6. Epub 2008/03/01. M708949200 [pii] 10.1074/jbc.M708949200 ; PubMed Central PMCID: PMC2442342. 18308731 PMC2442342

[101] Sijwali PS, Rosenthal PJ. Gene disruption confirms a critical role for the cysteine protease falcipain-2 in hemoglobin hydrolysis by Plasmodium falciparum. Proc Natl Acad Sci U S A. 2004;101(13):4384–9. 10.1073/pnas.0307720101 . 15070727 PMC384756

[102] Sijwali PS, Koo J, Singh N, Rosenthal PJ. Gene disruptions demonstrate independent roles for the four falcipain cysteine proteases of Plasmodium falciparum. Mol Biochem Parasitol. 2006;150(1):96–106. 10.1016/j.molbiopara.2006.06.013 . 16890302

[103] Blackman MJ. Malarial proteases and host cell egress: an 'emerging' cascade. Cell Microbiol. 2008;10(10):1925–34. 10.1111/j.1462-5822.2008.01176.x ; PubMed Central PMCID: PMCPMC2610400. 18503638 PMC2610400

[104] Collins CR, Hackett F, Atid J, Tan MSY, Blackman MJ. The Plasmodium falciparum pseudoprotease SERA5 regulates the kinetics and efficiency of malaria parasite egress from host erythrocytes. PLoS Pathog. 2017;13(7):e1006453 10.1371/journal.ppat.1006453 ; PubMed Central PMCID: PMCPMC5500368. 28683142 PMC5500368

[105] Ruecker A, Shea M, Hackett F, Suarez C, Hirst EM, Milutinovic K, et al Proteolytic activation of the essential parasitophorous vacuole cysteine protease SERA6 accompanies malaria parasite egress from its host erythrocyte. J Biol Chem. 2012;287(45):37949–63. 10.1074/jbc.M112.400820 ; PubMed Central PMCID: PMCPMC3488066. 22984267 PMC3488066

[106] Arastu-Kapur S, Ponder EL, Fonovic UP, Yeoh S, Yuan F, Fonovic M, et al Identification of proteases that regulate erythrocyte rupture by the malaria parasite Plasmodium falciparum. Nat Chem Biol. 2008;4(3):203–13. 10.1038/nchembio.70 . 18246061

[107] Ghosh S, Chisholm SA, Dans M, Lakkavaram A, Kennedy K, Ralph SA, et al The cysteine protease dipeptidyl aminopeptidase 3 does not contribute to egress of Plasmodium falciparum from host red blood cells. PLoS ONE. 2018;13(3):e0193538 10.1371/journal.pone.0193538 ; PubMed Central PMCID: PMCPMC5839547. 29509772 PMC5839547

[108] Thomas JA, Tan MSY, Bisson C, Borg A, Umrekar TR, Hackett F, et al A protease cascade regulates release of the human malaria parasite Plasmodium falciparum from host red blood cells. Nat Microbiol. 2018;3(4):447–55. 10.1038/s41564-018-0111-0 . 29459732 PMC6089347

[109] Greenbaum DC, Baruch A, Grainger M, Bozdech Z, Medzihradszky KF, Engel J, et al A role for the protease falcipain 1 in host cell invasion by the human malaria parasite. Science. 2002;298(5600):2002–6. 10.1126/science.1077426 . 12471262

[110] Eksi S, Czesny B, Greenbaum DC, Bogyo M, Williamson KC. Targeted disruption of Plasmodium falciparum cysteine protease, falcipain 1, reduces oocyst production, not erythrocytic stage growth. Mol Microbiol. 2004;53(1):243–50. 10.1111/j.1365-2958.2004.04108.x . 15225318

[111] Sijwali PS, Kato K, Seydel KB, Gut J, Lehman J, Klemba M, et al Plasmodium falciparum cysteine protease falcipain-1 is not essential in erythrocytic stage malaria parasites. Proc Natl Acad Sci U S A. 2004;101(23):8721–6. 10.1073/pnas.0402738101 ; PubMed Central PMCID: PMCPMC423262. 15166288 PMC423262

[112] Pandey KC, Singh N, Arastu-Kapur S, Bogyo M, Rosenthal PJ. Falstatin, a cysteine protease inhibitor of Plasmodium falciparum, facilitates erythrocyte invasion. PLoS Pathog. 2006;2(11):e117 10.1371/journal.ppat.0020117 ; PubMed Central PMCID: PMCPMC1630708. 17083274 PMC1630708

[113] Russo I, Oksman A, Goldberg DE. Fatty acid acylation regulates trafficking of the unusual Plasmodium falciparum calpain to the nucleolus. Mol Microbiol. 2009;72(1):229–45. 10.1111/j.1365-2958.2009.06639.x ; PubMed Central PMCID: PMCPMC2746569. 19239622 PMC2746569

[114] Russo I, Oksman A, Vaupel B, Goldberg DE. A calpain unique to alveolates is essential in Plasmodium falciparum and its knockdown reveals an involvement in pre-S-phase development. Proc Natl Acad Sci U S A. 2009;106(5):1554–9. 10.1073/pnas.0806926106 ; PubMed Central PMCID: PMCPMC2629787. 19164769 PMC2629787

[115] Datta G, Hossain ME, Asad M, Rathore S, Mohmmed A. Plasmodium falciparum OTU-like cysteine protease (PfOTU) is essential for apicoplast homeostasis and associates with noncanonical role of Atg8. Cell Microbiol. 2017;19(9):e12748 10.1111/cmi.12748 . 28423214

[116] Coppi A, Pinzon-Ortiz C, Hutter C, Sinnis P. The Plasmodium circumsporozoite protein is proteolytically processed during cell invasion. J Exp Med. 2005;201(1):27–33. 10.1084/jem.20040989 ; PubMed Central PMCID: PMCPMC1995445. 15630135 PMC1995445

[117] Hopp CS, Bennett BL, Mishra S, Lehmann C, Hanson KK, Lin JW, et al Deletion of the rodent malaria ortholog for falcipain-1 highlights differences between hepatic and blood stage merozoites. PLoS Pathog. 2017;13(9):e1006586 10.1371/journal.ppat.1006586 ; PubMed Central PMCID: PMCPMC5602738. 28922424 PMC5602738

[118] Eksi S, Czesny B, van Gemert GJ, Sauerwein RW, Eling W, Williamson KC. Inhibition of Plasmodium falciparum oocyst production by membrane-permeant cysteine protease inhibitor E64d. Antimicrob Agents Chemother. 2007;51(3):1064–70. 10.1128/AAC.01012-06 ; PubMed Central PMCID: PMCPMC1803139. 17178799 PMC1803139

[119] Suarez-Cortes P, Sharma V, Bertuccini L, Costa G, Bannerman NL, Sannella AR, et al Comparative Proteomics and Functional Analysis Reveal a Role of Plasmodium falciparum Osmiophilic Bodies in Malaria Parasite Transmission. Mol Cell Proteomics. 2016;15(10):3243–55. 10.1074/mcp.M116.060681 ; PubMed Central PMCID: PMCPMC5054347. 27432909 PMC5054347

[120] Dahl EL, Rosenthal PJ. Biosynthesis, localization, and processing of falcipain cysteine proteases of Plasmodium falciparum. Mol Biochem Parasitol. 2005;139(2):205–12. Epub 2005/01/25. S0166-6851(04)00313-5 [pii] 10.1016/j.molbiopara.2004.11.009 . 15664655

[121] Shenai BR, Sijwali PS, Singh A, Rosenthal PJ. Characterization of native and recombinant falcipain-2, a principal trophozoite cysteine protease and essential hemoglobinase of Plasmodium falciparum. J Biol Chem. 2000;275(37):29000–10. 10.1074/jbc.M004459200 . 10887194

[122] Singh N, Sijwali PS, Pandey KC, Rosenthal PJ. Plasmodium falciparum: biochemical characterization of the cysteine protease falcipain-2'. Exp Parasitol. 2006;112(3):187–92. Epub 2005/12/13. S0014-4894(05)00280-8 [pii] 10.1016/j.exppara.2005.10.007 . 16337629

[123] Sijwali PS, Shenai BR, Rosenthal PJ. Folding of the Plasmodium falciparum cysteine protease falcipain-2 is mediated by a chaperone-like peptide and not the prodomain. J Biol Chem. 2002;277(17):14910–5. Epub 2002/02/06. 10.1074/jbc.M109680200 [pii]. . 11827964

[124] Marco M, Coteron JM. Falcipain inhibition as a promising antimalarial target. Curr Top Med Chem. 2012;12(5):408–44. . 22242849 10.2174/156802612799362913

[125] Kerr ID, Lee JH, Pandey KC, Harrison A, Sajid M, Rosenthal PJ, et al Structures of falcipain-2 and falcipain-3 bound to small molecule inhibitors: implications for substrate specificity. J Med Chem. 2009;52(3):852–7. Epub 2009/01/09. 10.1021/jm8013663 [pii]. ; PubMed Central PMCID: PMC2651692. 19128015 PMC2651692

[126] Olson JE, Lee GK, Semenov A, Rosenthal PJ. Antimalarial effects in mice of orally administered peptidyl cysteine protease inhibitors. Bioorg Med Chem. 1999;7(4):633–8. . 10353642 10.1016/s0968-0896(99)00004-8

[127] Coteron JM, Catterick D, Castro J, Chaparro MJ, Diaz B, Fernandez E, et al Falcipain inhibitors: optimization studies of the 2-pyrimidinecarbonitrile lead series. J Med Chem. 2010;53(16):6129–52. 10.1021/jm100556b . 20672841

[128] Conroy T, Guo JT, Elias N, Cergol KM, Gut J, Legac J, et al Synthesis of gallinamide A analogues as potent falcipain inhibitors and antimalarials. J Med Chem. 2014;57(24):10557–63. 10.1021/jm501439w . 25412465

[129] Melo PMS, El Chamy Maluf S, Azevedo MF, Paschoalin T, Budu A, Bagnaresi P, et al Inhibition of Plasmodium falciparum cysteine proteases by the sugarcane cystatin CaneCPI-4. Parasitol Int. 2018;67(2):233–6. 10.1016/j.parint.2017.12.005 . 29288140

[130] Singh A, Rosenthal PJ. Selection of cysteine protease inhibitor-resistant malaria parasites is accompanied by amplification of falcipain genes and alteration in inhibitor transport. J Biol Chem. 2004;279(34):35236–41. 10.1074/jbc.M404235200 . 15192087

[131] Klonis N, Crespo-Ortiz MP, Bottova I, Abu-Bakar N, Kenny S, Rosenthal PJ, et al Artemisinin activity against Plasmodium falciparum requires hemoglobin uptake and digestion. Proc Natl Acad Sci U S A. 2011;108(28):11405–10. 10.1073/pnas.1104063108 ; PubMed Central PMCID: PMC3136263. 21709259 PMC3136263

[132] Semenov A, Olson JE, Rosenthal PJ. Antimalarial synergy of cysteine and aspartic protease inhibitors. Antimicrob Agents Chemother. 1998;42(9):2254–8. . 9736544 10.1128/aac.42.9.2254 PMC105801

[133] Robert-Gangneux F, Darde ML. Epidemiology of and diagnostic strategies for toxoplasmosis. Clin Microbiol Rev. 2012;25(2):264–96. 10.1128/CMR.05013-11 ; PubMed Central PMCID: PMCPMC3346298. 22491772 PMC3346298

[134] Dou Z, McGovern OL, Di Cristina M, Carruthers VB. Toxoplasma gondii ingests and digests host cytosolic proteins. MBio. 2014;5(4):e01188–14. 10.1128/mBio.01188-14 ; PubMed Central PMCID: PMCPMC4161261. 25028423 PMC4161261

[135] Larson ET, Parussini F, Huynh MH, Giebel JD, Kelley AM, Zhang L, et al Toxoplasma gondii cathepsin L is the primary target of the invasion-inhibitory compound morpholinurea-leucyl-homophenyl-vinyl sulfone phenyl. J Biol Chem. 2009;284(39):26839–50. 10.1074/jbc.M109.003780 ; PubMed Central PMCID: PMCPMC2785372. 19596863 PMC2785372

[136] Teo CF, Zhou XW, Bogyo M, Carruthers VB. Cysteine protease inhibitors block Toxoplasma gondii microneme secretion and cell invasion. Antimicrob Agents Chemother. 2007;51(2):679–88. 10.1128/AAC.01059-06 ; PubMed Central PMCID: PMCPMC1797762. 17145790 PMC1797762

[137] Que X, Engel JC, Ferguson D, Wunderlich A, Tomavo S, Reed SL. Cathepsin Cs are key for the intracellular survival of the protozoan parasite, Toxoplasma gondii. J Biol Chem. 2007;282(7):4994–5003. 10.1074/jbc.M606764200 . 17164247

[138] Di Cristina M, Dou Z, Lunghi M, Kannan G, Huynh MH, McGovern OL, et al Toxoplasma depends on lysosomal consumption of autophagosomes for persistent infection. Nat Microbiol. 2017;2:17096 10.1038/nmicrobiol.2017.96 ; PubMed Central PMCID: PMCPMC5527684. 28628099 PMC5527684

[139] Tillack M, Biller L, Irmer H, Freitas M, Gomes MA, Tannich E, et al The Entamoeba histolytica genome: primary structure and expression of proteolytic enzymes. BMC Genomics. 2007;8:170 10.1186/1471-2164-8-170 ; PubMed Central PMCID: PMCPMC1913524. 17567921 PMC1913524

[140] Que X, Ngo H, Lawton J, Gray M, Liu Q, Engel J, et al The cathepsin B of Toxoplasma gondii, toxopain-1, is critical for parasite invasion and rhoptry protein processing. J Biol Chem. 2002;277(28):25791–7. 10.1074/jbc.M202659200 . 12000756

[141] Huang R, Que X, Hirata K, Brinen LS, Lee JH, Hansell E, et al The cathepsin L of Toxoplasma gondii (TgCPL) and its endogenous macromolecular inhibitor, toxostatin. Mol Biochem Parasitol. 2009;164(1):86–94. 10.1016/j.molbiopara.2008.11.012 ; PubMed Central PMCID: PMCPMC2663568. 19111576 PMC2663568

[142] Que X, Wunderlich A, Joiner KA, Reed SL. Toxopain-1 is critical for infection in a novel chicken embryo model of congenital toxoplasmosis. Infect Immun. 2004;72(5):2915–21. 10.1128/IAI.72.5.2915-2921.2004 ; PubMed Central PMCID: PMCPMC387868. 15102804 PMC387868

[143] Dou Z, Coppens I, Carruthers VB. Non-canonical maturation of two papain-family proteases in Toxoplasma gondii. J Biol Chem. 2013;288(5):3523–34. 10.1074/jbc.M112.443697 ; PubMed Central PMCID: PMCPMC3561571. 23250753 PMC3561571

[144] Chaparro JD, Cheng T, Tran UP, Andrade RM, Brenner SBT, Hwang G, et al Two key cathepsins, TgCPB and TgCPL, are targeted by the vinyl sulfone inhibitor K11777 in in vitro and in vivo models of toxoplasmosis. PLoS ONE. 2018;13(3):e0193982 10.1371/journal.pone.0193982 ; PubMed Central PMCID: PMCPMC5863946. 29565998 PMC5863946

[145] Nguyen HM, El Hajj H, El Hajj R, Tawil N, Berry L, Lebrun M, et al Toxoplasma gondii autophagy-related protein ATG9 is crucial for the survival of parasites in their host. Cell Microbiol. 2017;19(6):e12712 10.1111/cmi.12712 . 27992947

[146] Zhao G, Zhou A, Lv G, Meng M, Sun M, Bai Y, et al Toxoplasma gondii cathepsin proteases are undeveloped prominent vaccine antigens against toxoplasmosis. BMC Infect Dis. 2013;13:207 10.1186/1471-2334-13-207 ; PubMed Central PMCID: PMCPMC3659040. 23651838 PMC3659040

[147] Han Y, Zhou A, Lu G, Zhao G, Sha W, Wang L, et al DNA Vaccines Encoding Toxoplasma gondii Cathepsin C 1 Induce Protection against Toxoplasmosis in Mice. Korean J Parasitol. 2017;55(5):505–12. 10.3347/kjp.2017.55.5.505 ; PubMed Central PMCID: PMCPMC5678475. 29103265 PMC5678475

[148] Grab DJ, Garcia-Garcia JC, Nikolskaia OV, Kim YV, Brown A, Pardo CA, et al Protease activated receptor signaling is required for African trypanosome traversal of human brain microvascular endothelial cells. PLoS Negl Trop Dis. 2009;3(7):e479 10.1371/journal.pntd.0000479 ; PubMed Central PMCID: PMCPMC2707606. 19621073 PMC2707606

[149] Mahmoudzadeh-Niknam H, McKerrow JH. Leishmania tropica: cysteine proteases are essential for growth and pathogenicity. Exp Parasitol. 2004;106(3–4):158–63. Epub 2004/06/03. 10.1016/j.exppara.2004.03.005 . 15172223

[150] Besteiro S, Williams RA, Morrison LS, Coombs GH, Mottram JC. Endosome sorting and autophagy are essential for differentiation and virulence of Leishmania major. J Biol Chem. 2006;281(16):11384–96. 10.1074/jbc.M512307200 . 16497676

[151] De Souza Leao S, Lang T, Prina E, Hellio R, Antoine JC. Intracellular Leishmania amazonensis amastigotes internalize and degrade MHC class II molecules of their host cells. J Cell Sci. 1995;108(Pt 10):3219–31. . 7593283 10.1242/jcs.108.10.3219

[152] Abdulla MH, O'Brien T, Mackey ZB, Sajid M, Grab DJ, McKerrow JH. RNA interference of Trypanosoma brucei cathepsin B and L affects disease progression in a mouse model. PLoS Negl Trop Dis. 2008;2(9):e298 Epub 2008/09/30. 10.1371/journal.pntd.0000298 ; PubMed Central PMCID: PMC2553486. 18820745 PMC2553486

[153] Garcia MP, Nobrega OT, Teixeira AR, Sousa MV, Santana JM. Characterisation of a Trypanosoma cruzi acidic 30 kDa cysteine protease. Mol Biochem Parasitol. 1998;91(2):263–72. . 9566519 10.1016/s0166-6851(97)00205-3

[154] Tolbert MK, Brand MD, Gould EN. In vitro effects of cysteine protease inhibitors on Trichomonas foetus-induced cytopathic changes in porcine intestinal epithelial cells. Am J Vet Res. 2016;77(8):890–7. 10.2460/ajvr.77.8.890 . 27463553

[155] Cobo ER, Reed SL, Corbeil LB. Effect of vinyl sulfone inhibitors of cysteine proteinases on Tritrichomonas foetus infection. Int J Antimicrob Agents. 2012;39(3):259–62. 10.1016/j.ijantimicag.2011.09.026 ; PubMed Central PMCID: PMCPMC3279618. 22104282 PMC3279618

[156] Chen YT, Brinen LS, Kerr ID, Hansell E, Doyle PS, McKerrow JH, et al In vitro and in vivo studies of the trypanocidal properties of WRR-483 against Trypanosoma cruzi. PLoS Negl Trop Dis. 2010;4(9):e825 10.1371/journal.pntd.0000825 ; PubMed Central PMCID: PMCPMC2939063. 20856868 PMC2939063

[157] Stolze SC, Deu E, Kaschani F, Li N, Florea BI, Richau KH, et al The antimalarial natural product symplostatin 4 is a nanomolar inhibitor of the food vacuole falcipains. Chem Biol. 2012;19(12):1546–55. 10.1016/j.chembiol.2012.09.020 ; PubMed Central PMCID: PMCPMC3601557. 23261598 PMC3601557

[158] Brak K, Kerr ID, Barrett KT, Fuchi N, Debnath M, Ang K, et al Nonpeptidic tetrafluorophenoxymethyl ketone cruzain inhibitors as promising new leads for Chagas disease chemotherapy. J Med Chem. 2010;53(4):1763–73. 10.1021/jm901633v ; PubMed Central PMCID: PMCPMC2838180. 20088534 PMC2838180

[159] Gauthier JY, Chauret N, Cromlish W, Desmarais S, Duong LT, Falgueyret JP, et al The discovery of odanacatib (MK-0822), a selective inhibitor of cathepsin K. Bioorg Med Chem Lett. 2008;18(3):923–8. 10.1016/j.bmcl.2007.12.047 . 18226527

[160] Duong le T, Leung AT, Langdahl B. Cathepsin K Inhibition: A New Mechanism for the Treatment of Osteoporosis. Calcif Tissue Int. 2016;98(4):381–97. 10.1007/s00223-015-0051-0 . 26335104

[161] Brumatti G, Ma C, Lalaoui N, Nguyen NY, Navarro M, Tanzer MC, et al The caspase-8 inhibitor emricasan combines with the SMAC mimetic birinapant to induce necroptosis and treat acute myeloid leukemia. Sci Transl Med. 2016;8(339):339ra69 10.1126/scitranslmed.aad3099 . 27194727

[162] McKerrow JH. Development of cysteine protease inhibitors as chemotherapy for parasitic diseases: insights on safety, target validation, and mechanism of action. Int J Parasitol. 1999;29(6):833–7. . 10480720 10.1016/s0020-7519(99)00044-2

[163] McKerrow JH, Caffrey C, Kelly B, Loke P, Sajid M. Proteases in parasitic diseases. Annu Rev Pathol. 2006;1:497–536. 10.1146/annurev.pathol.1.110304.100151 . 18039124

